# Timing of maternal exposure to toxic cyanobacteria and offspring fitness in *Daphnia magna*: Implications for the evolution of anticipatory maternal effects

**DOI:** 10.1002/ece3.4700

**Published:** 2018-11-20

**Authors:** Reinder Radersma, Alexander Hegg, Daniel W. A. Noble, Tobias Uller

**Affiliations:** ^1^ Department of Biology Lund University Lund Sweden; ^2^ Ecology and Evolution Research Centre, School of Biological, Earth and Environmental Sciences The University of New South Wales Sydney New South Wales Australia

**Keywords:** egg size, fitness, microcystin, parental effects, stress, tolerance, transgenerational effects

## Abstract

Organisms that regularly encounter stressful environments are expected to use cues to develop an appropriate phenotype. Water fleas (*Daphnia* spp.) are exposed to toxic cyanobacteria during seasonal algal blooms, which reduce growth and reproductive investment. Because generation time is typically shorter than the exposure to cyanobacteria, maternal effects provide information about the local conditions subsequent generations will experience. Here, we evaluate if maternal effects in response to microcystin, a toxin produced by cyanobacteria, represent an inheritance system evolved to transmit information in *Daphnia magna*. We exposed mothers as juveniles and/or as adults, and tested the offspring's fitness in toxic and non‐toxic environments. Maternal exposure until reproduction reduced offspring fitness, both in the presence and in the absence of toxic cyanobacteria. However, this effect was accompanied by a small positive fitness effect, relative to offspring from unexposed mothers, in the presence of toxic cyanobacteria. This effect was mainly elicited in response to maternal exposure to toxic cyanobacteria early in life and less so during reproduction. None of these effects were explained by changes in egg size. A meta‐analysis using our and others’ experiments suggests that the adaptive value of maternal effects to cyanobacteria exposure is weak at best. We suggest that the beneficial maternal effect in our study is an example of phenotypic accommodation spanning generations, rather than a mechanism evolved to transmit information about cyanobacteria presence between generations.

## 1 INTRODUCTION

Many organisms regularly encounter conditions that are harmful to their survival or reproduction. This makes it beneficial to be responsive to cues that allow the development of an appropriate phenotype to cope with stressful conditions. The phenotype of the mother is sometimes an informative cue, in particular, if plastic responses in parents correlate with selective regimes experienced by offspring (English, Pen, Shea, & Uller, 2015; McNamara, Dall, Hammerstein, & Leimar, 2016; Uller, 2008). This is a likely reason for the many instances of maternally determined diapause, dormancy, and other forms of adaptive plasticity in seasonal environments (Donohue, 2009; Mousseau & Fox, 1998; Tauber, Tauber, & Masaki, 1986). Such adaptive, context‐dependent, maternal effects are often referred to as “anticipatory maternal effects” or “adaptive transgenerational plasticity” (Marshall & Uller, 2007).

Not all parental effects are beneficial, however. Females that reproduce under stressful conditions may provide insufficient resources for their offspring or disrupt their development in other ways, for example, via passive transmission of toxins to the egg or across the placenta (Crump & Trudeau, 2009; Schwindt, 2015; Smith et al., 2007; Tsui & Wang, 2004). Such changes in resource provisioning or non‐adaptive transfer of harmful compounds nevertheless carry information about the local environment. Furthermore, exposure to stress during early development can allow individuals to cope better with similar conditions later in life (Badyaev, 2005b; Huether, 1996).

A plausible evolutionary scenario is that anticipatory maternal effects evolve by modification of responses that originally were “passive” consequences of changes to maternal–offspring interactions under stressful conditions (Badyaev, 2005b; Badyaev & Uller, 2009). If conditions are sufficiently recurrent, natural selection should reduce negative effects and strengthen positive effects. This may eventually lead to the evolution of a “detection‐based” inheritance systems (sensu Shea, Pen, & Uller, 2011) by which parental responses to the local environment allow reliable transmission of information without imposing costs on the offspring (Badyaev & Uller, 2009; McNamara et al., 2016; Uller, 2012).

Water fleas (*Daphnia* spp.) inhabit water bodies which vary seasonally in biotic (e.g., community structure) and abiotic features (e.g., climatic variables). Adaptive plasticity is well characterized in many species, including morphological and physiological responses that increase survival under predation (Krueger & Dodson, 1981), parasitism (Chadwick & Little, 2005), UV exposure (Rhode, Pawlowski, & Tollrian, 2001), extreme temperatures (Henning‐Lucass, Cordellier, Streit, & Schwenk, 2016), and toxins (von Elert, Zitt, & Schwarzenberger, 2012). Maternal exposure to several of these stressors has also been shown to occasionally increase the fitness of their offspring if those offspring encounter the same conditions (e.g., inducible defences; Agrawal, Laforsch, & Tollrian, 1999).

One putative example is the seasonal exposure to toxic cyanobacteria during algal blooms, which has been tested for a small number of clones with inconsistent results (Gustafsson, Rengefors, & Hansson, 2005; Hansson, Gustafsson, Rengefors, & Bomark, 2007; Ortiz‐Rodriguez, Dao, & Wiegand, 2012). Algal blooms often persist for several generations (Hansson et al., 2007), but resistant genotypes may not go to fixation if tolerance is physiologically costly during the part of the year when cyanobacteria are at low density. Thus, regular seasonal exposure to cyanobacteria appears to fulfill conditions that select for a mechanism enabling mothers to transmit information about this feature of the environment to their offspring (English et al., 2015; McNamara et al., 2016; Uller, English, & Pen, 2015). However, it is unclear if maternal effects to cyanobacteria exhibit the features we might expect for an inheritance system designed to enable information about environmental toxicity to be effectively communicated from one generation to the next.

The efficacy of information transfer to offspring can be difficult to assess (Uller, Nakagawa, & English, 2013). For example, specific responses to the information carried by the maternal phenotype may be obscured by non‐additive direct effects of the maternal and the offspring environments (Engqvist & Reinhold, 2016; Nettle & Bateson, 2015). It is therefore useful to experimentally separate the negative carry‐over effects from any adaptive mechanism of information transfer between generations. To better understand to what extent *Daphnia* have evolved to transfer information about the presence of toxic cyanobacteria via maternal effects, we designed a series of experiments that manipulated maternal and offspring exposure to cyanobacteria that either produce or do not produce microcystin, one of the main toxins present in cyanobacteria. Since several unknown mechanisms could be responsible for maternal effects, we could not experimentally isolate the fitness value of information per se (sensu Donaldson‐Matasci, Bergstrom, & Lachmann, 2010) from non‐additive effects of maternal and offspring environments (Engqvist & Reinhold, 2016). However, since we expected that growth and reproduction in the presence of toxic cyanobacteria would be compromised, we exposed mothers either throughout their life, only before the onset of reproduction, or only after the onset of reproduction.

The rationale for this design is that a maternal effect that has evolved into a “channel of inheritance” should limit the negative effects of growing up under stressful conditions, because mothers would buffer their offspring from stress. Furthermore, a mechanism that enables adaptive transfer of information about the presence of toxic cyanobacteria should be active at a time when the maternal assessment of the environment that will be encountered by offspring is most likely to be accurate, which in this case is during reproduction (but see Taborsky, 2006). Accordingly, if maternal effects have evolved to transmit information, we expect that there should not only be a positive effect of matching maternal and offspring environments, but that this effect should be strongest when mothers were exposed only during reproduction (i.e., sending information about the local environment to offspring without offspring paying any cost associated with prolonged maternal exposure to toxin). Alternatively, a positive effect on offspring tolerance may be a spill‐over effect of physiological changes in the mother that primarily serve to protect her from the toxin, and not to transmit information (Badyaev, 2005a)*.* This would be supported if offspring are severely negatively affected by maternal exposure, and any positive effect would be consistent with passing on tolerance built up through prolonged maternal exposure, passively.

## 2 MATERIALS AND METHODS

**Figure 1:**
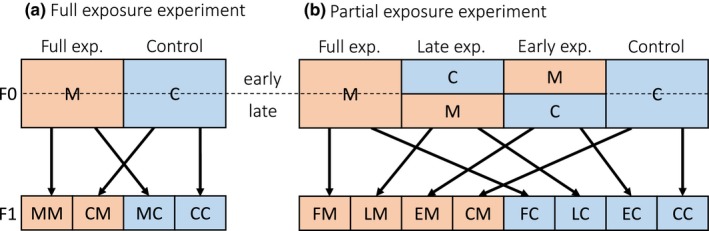
Experimental design for (a) the full maternal exposure experiments (1, 2 and 3) and (b) the partial maternal exposure experiments (4 and 5). M is the toxic microcystin treatment and C is control treatment. For the second generation (F1), the first letter is the maternal (F0) treatment and the second letter the offspring (F1) treatment. In the partial exposure experiments, “F,” “L,” “E,” and “C” indicate maternal full, late, early, and control treatment respectively. For instance, CM means that the mother was in the control treatment, while her offspring was in the microcystin treatment

In April and May 2015, we isolated *Daphnia magna* from Lake Bysjön (surface area 10 ha, 55°40'32"N 13°32'42"E) in Southern Sweden, a eutrophic lake that frequently develops algae blooms in some but not all years (Gustafsson, 2007; Schwarzenberger, D'Hondt, Vyverman, & Elert, 2013). Fifteen genetically unique clone lines (determined with six microsatellites) were kept reproducing asexually for 2 months until the start of the first experiment. Over a period of 2 years, we performed five experiments. In three experiments, we tested for maternal effects in response to exposure to cyanobacteria which either produced or did not produce the toxin microcystin using a two‐treatment design (Figure 1a; called *full exposure experiments* from this point onwards). Moreover, in two experiments, we investigated how the timing of maternal exposure influenced the strength of the maternal effect using a four‐treatment design (Figure 1b; called *partial exposure experiments* from this point onwards). The replication of the experiments ensures that the total sample size allowed detection of subtle maternal effects (see Table 1) and increased the reliability and robustness of our findings relative to previous studies, most of which relied on a single clone (see Table 2). More detailed descriptions of the design of these experiments are found below.

**Table 1:** Overview of the experiments exposing two generations of *Daphnia magna* to cyanobacteria that produced microcystin (“toxic treatment”) or to cyanobacteria that did not produce microcystin (“control treatment”)

Experiment	Start date	End date	Toxicity (μg/L; ± *SE*)	*N*_C_	Generation 1			Generation 2		
					*N*_E.1_	*N*_R.1_	*N*_I.1_	*N*_E.2_	*N*_R.2_	*N*_I.2_
1. Full exposure	08/07/2015	27/08/2015	C: 0.0964 ± 0.00190 M: 5.186 ± 0.738	15	2	4	61 (120)	2×2	4	68 (240)
2. Full exposure	13/05/2016	15/08/2016	C: 0.0525 ± 0.0110 M: 0.856 ± 0.0965	5	2	4	37 (40)	2×2	4	71 (80)
3. Full exposure	09/11/2016	23/01/2017	C: 0.0200 ± 0.00601 M: 0.633 ± 0.0614	5	2	3	46 (30)	2×2	3	50 (60)
4. Partial exposure	10/07/2016	08/09/2016	C: 0.0252 ± 0.0110 M: 0.511 ± 0.147	7	4	3x2	130 (151)	4×2	3×3	408 (456)
5 Partial exposure	08/05/2017	23/07/2017	C: 0.0243 ± 0.0101 M: 0.396 ± 0.0195	7	4	3x2	111 (168)	4×2	3×3	461 (504)

Note

Experiments 1–3 exposed mothers for the full duration of their lives, whereas experiments 4 and 5 had four maternal treatment groups (control, early, late, and full exposure of generation 1). All experiments were factorial, with the offspring always being exposed for the full duration of their life. Toxicity is the microcystin concentration in the control (“C”) and toxic treatment (“M”). Number of clone lines (*N*_C_) and—for both generations separately—the number of experimental treatments (*N*_E_), replicates (*N*_R_), and the realized samples sizes (i.e., for which all data is available; *N*_I_). In brackets are the anticipated sample sizes if all individuals would have survived until all data could be collected.

**Table 2:** Overview of the studies used in the meta‐analysis

Study	Species	*N*_T_	Toxicity (μg/L)	*N*_C_	*N*_CM_	*N*_MM_
Gustafsson et al. (2005)	*Daphnia magna*	4	0.88	1	31	31
Lyu et al. (2016)	*D. magna*	3	3.64	2	20	20
Jiang et al. (2013)	*Daphnia carinata*	5	4.82	3	90	90
Ortiz‐Rodriguez et al. (2012)	*D. magna*	8	3.09–21.75	1	20	20
Dao et al. (2010)	*D. magna*	2	5–50	1	100	100
Brett (1993)	*Daphnia longispina*	8	n/a	1	22–48	22–47
Experiment 1	*D. magna*	5	5.186	10[Link]	5–20	6–15
Experiment 2	*D. magna*	7	0.856	4[Link]	16	15
Experiment 3	*D. magna*	7	0.633	2[Link]	10	7–9
Experiment 4	*D. magna*	7	0.511	7	51	48
Experiment 5	*D. magna*	7	0.396	7	61	53

Note

There are six published studies and five of our own experiments. *N*_T_ indicates the number of measured traits. Toxicity is the mean concentration of microcystin in the toxic treatment reported for the studies (multiple concentrations are indicated by a range). *N*_C_ is the number of clones. *N*_CM_ is the sample size for offspring in the toxic treatment, when the mother was not exposed and *N*_MM_ is the sample size for offspring in the toxic treatment, when the mother was also exposed (different sample sizes for different traits are indicated by a range).

For some of the clones, measurements on offspring in both treatments were not available.

### 2.1 Laboratory practice and experimental design

The *Daphnia magna* clone lines were kept in 1 L jars at 18°C, with a 14:10 light:dark cycle and fed with *Scenedesmus obliquus* (strain: NIVA CHL‐6). Prior to each experiment, we isolated individuals from the clone lines and kept them individually for at least 2 generations in 100 ml jars with artificial lake water (Klüttgen, Dülmer, Engels, & Ratte, 1994) and 120,000 cells/ml of green algae. We used offspring of these isolated individuals for the experiments. During the experiments, all animals were kept individually in 100 ml jars with artificial lake water, 120,000 cells/ml of green algae and 280,000 cells/ml (experiment 1), 70,000 cells/ml (experiment 2 and 3), or 35,000 cells/ml (experiment 4 and 5) of cyanobacteria. The concentrations of cyanobacteria followed previous studies of maternal effects in this population (Gustafsson et al., 2005). Depending on the treatment, these cyanobacteria were a strain producing the toxin microcystin, *Microcystis aeruginosa* (NIVA CYA‐228/1) hereafter denoted *microcystin treatment* (*M*), or a strain not producing microcystin, *Microcystis aeruginosa* (NIVA CYA‐143) hereafter denoted *control treatment* (*C*). Both strains have similarly sized cells and did not show any colony formation during the experiments. Since our purpose was to test the response to the toxin microcystin, we refer to the cyanobacteria strains as toxic and non‐toxic, respectively, even if both strains also produce other compounds that can be toxic to *Daphnia* (Schwarzenberger & Fink, 2018). Toxicity levels were measured on medium samples collected during the experiments with an enzyme‐linked immunosorbent assay for the congener‐independent determination of microcystins and nodularins (Abraxis Microcystins‐ADDA ELISA kit) according to the manufacturer's specifications. The difference in cell density between experiments is accompanied by differences in toxicity of the treatment (Table 1). Water was changed every other day. Individuals were checked daily and the first two broods were counted and removed and the third brood was counted and used to populate the next generation if necessary (in experiment 1, the second brood was used to populate the next generation because of low survival as a result of high toxicity; Table 1). We estimated fitness by calculating, for each individual, the intrinsic rate of population increase *r* (the rate at which an individual replaces itself) with a univariate root finding algorithm (uniroot in *R*) using the Euler equation:

(1) $$1=e^{-ra_{1}}b_{1}+e^{-ra_{2}}b_{2}+e^{-ra_{3}}b_{3}$$

where *a_i_* is the age of reproduction at reproductive event *i* and *b_i_* is the brood size produced at *a_i_*. For experiment 1, we applied the formula without the last term. Only individuals which produced at least three broods (or two broods for experiment 1) were included in the analyses (see Table 1 for sample sizes). Since the number of offspring is a commonly used proxy for fitness, we repeated this analysis for the total number of offspring produced in the first three broods (or two broods for experiment 1).

The full exposure experiments were performed in a fully factorial design. Mothers were exposed their whole life either to the toxic (i.e., full) or to the non‐toxic (i.e., control) cyanobacteria. The third brood (second in experiment 1) offspring were split over the same treatments, with at least 3 offspring per mother in each treatment (Figure 1a). The partial exposure experiments were similar to the full exposure experiments, but in addition to maternal exposure for the duration of her life, we also exposed mothers to toxic cyanobacteria either only up until the first reproductive event (i.e., early) or only as an adult (i.e., late). We defined the early treatment up until the release of her first brood and late treatment after this point. The offspring were split over the full and control treatments, with at least three offspring per mother in each treatment (Figure 1b).

From experiment 3 and 4, we collected the individuals after they released their third brood and stored them in 70% ethanol to measure egg sizes. Using an Olympus SZX10 stereo microscope, we dissected eggs from the fourth brood out of the mothers’ carapace. We photographed the eggs with a mounted Olympus SC50 digital camera and used Olympus cellSens Standard (version 1.15) to measure the size of the eggs by calculating the area of the eggs by using its ellipse drawing tool. For experiment 3, we had 20 mothers from the control treatment and 21 mothers from the toxic treatment in both the first and second generation, resulting in a total of 138 and 86 egg size measurements, respectively. For experiment 4, we had 22 mothers from the control treatment and 19 mothers from the toxic treatment from only the second generation, resulting in a total of 132 and 63 egg size measurements, respectively.

### 2.2 Data analysis

**Figure 2:**
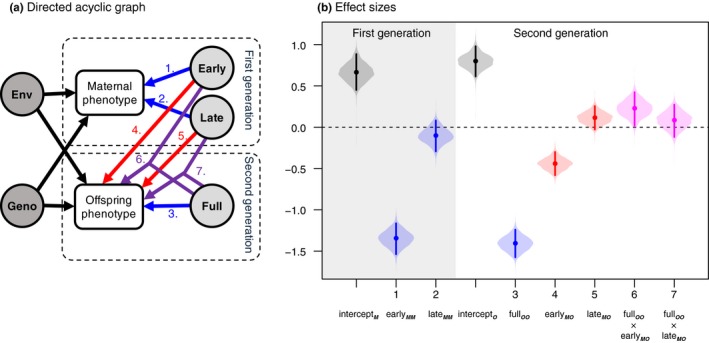
Directed acyclic graph of (a) the statistical model and (b) and the corresponding effect sizes. Colored arrows in (a) correspond to posterior estimates presented in (b). Dots are the means, whiskers are the 95% credible intervals and violins are the distributions of the posterior estimates. On the gray background are the effects on maternal fitness (first generation, marked with [second] subscript M) and on the white background are the effects on the offspring fitness (second generation, marked with [second] subscript O). In blue are the estimates for direct treatment effects (marked with MM or OO, the effect of maternal exposure on maternal fitness and the effect of offspring exposure on offspring fitness respectively), in red are the estimates for maternal effects (marked with subscript MO) and in purple are the interactions of the maternal effects with the offspring environment

To analyze the partial exposure experiments, we constructed two maternal effects models with their own respective outcome variables; maternal and offspring fitness. We modeled maternal and offspring fitness using general linear mixed models with their own sets of explanatory variables; however, genotype effects and the distribution of error terms were shared. We explained maternal fitness with early and late exposure. We explained offspring fitness with early and late exposure of the mother, full exposure of the offspring and the interactions between early maternal exposure and full offspring exposure and late maternal exposure and full offspring exposure (Figure 2a). We modeled all combinations of explanatory variables and cross‐validated those models with Watanabe‐Akaike information criterion and approximate leave‐one‐out cross‐validation (Vehtari, Gelman, & Gabry, 2017). Because many of the models performed similarly and all explanatory variables were present in the best performing models (Table S1), we present only the full model in the main text. Models were analyzed with *Stan,* a Bayesian general‐purpose C++ inference library (Carpenter et al., 2017). The results for the total number of offspring produced are presented in Table S2 and Figure S1 and are similar to the results of fitness estimated as *r*.

We analyzed egg size with a general linear mixed model in which we explained egg size by a best linear unbiased prediction (BLUP) for each mother. Simultaneously, these maternal average egg sizes were explained by exposure to toxic cyanobacteria, clutch interval (measured as the time difference in the release of the first and the third brood divided by 2), the average size of the second and third clutch and clone effects. We introduced clutch interval in the model to correct for any potential effects of development rate and therefore development state on the egg size measurements, since offspring develop slower for mothers in the microcystin treatment compared to the control. We used the maternal average egg size in a model of offspring fitness together with offspring treatment (Figure 4a). Explaining offspring fitness with egg size directly was not possible because we do not have egg size measurements for hatched offspring. The model was analyzed with *Stan*.

### 2.3 Generality of the results: A meta‐analysis

To test the robustness of any positive maternal effects, we further performed a meta‐analysis using our five experiments and published studies. On March 28, 2017, we searched Web of Science for studies using the search terms “Daphnia AND Microcystin” in titles, abstracts, and keywords, resulting in 211 hits. We scanned titles and abstracts for experimental studies investigating maternal effects, narrowing the selection to 20 papers. We forward and backwards searched citing articles and the citations of these 20 papers, resulting in one additional paper of interest. We collected data from six papers where the maternal generation was exposed to *Microcystis* spp. or microcystin and the offspring to the same and a control treatment (note that none of the previous studies have been designed to isolate the information value of maternal effects, i.e., they all represent treatments similar to our “maternal full exposure”). The selected studies are listed in Table 2. From these studies, we extracted, for each genotype individually, statistics on traits that can be seen as fitness estimates, components or proxies, such as replacement rates, ages of reproduction, brood sizes, survival estimates, and body size measurements. We calculated Hedges’ g for the effect of maternal exposure on the estimate of offspring fitness only when offspring were themselves exposed (i.e., we compared offspring in a toxic treatment that were born to mothers in a toxic treatment to offspring in a toxic treatment born to mothers in a control treatment). Hedges’ *g* was calculated from the means and corresponding standard deviations (which in some cases were calculated from standard errors). In the case of survival estimates, we converted the log odds ratio to Hedges’ *g* (calculated from the survival probabilities and sample sizes).

For the meta‐analysis, we used a multi‐level meta‐analytic model (Nakagawa & Santos, 2012). In our models, we estimated between‐study effects, between‐clone effects, trait class effects, and within‐study effects. We classified traits as being a fitness estimate, offspring quantity measurement, age of reproduction measurement, survival estimate or morphometric measurement, and treated these classes as a random effect. For the studies reporting toxicity levels, we also ran the model with the concentration of microcystin as an explanatory variable to investigate whether this affected the strength of the maternal effects. The models were analyzed with *Stan*. Code and more details on the models, inference practices, and cross‐validation for each analysis can be found in Appendices S1–S5.

## 3 RESULTS

### 3.1 Maternal effects

**Figure 3:**
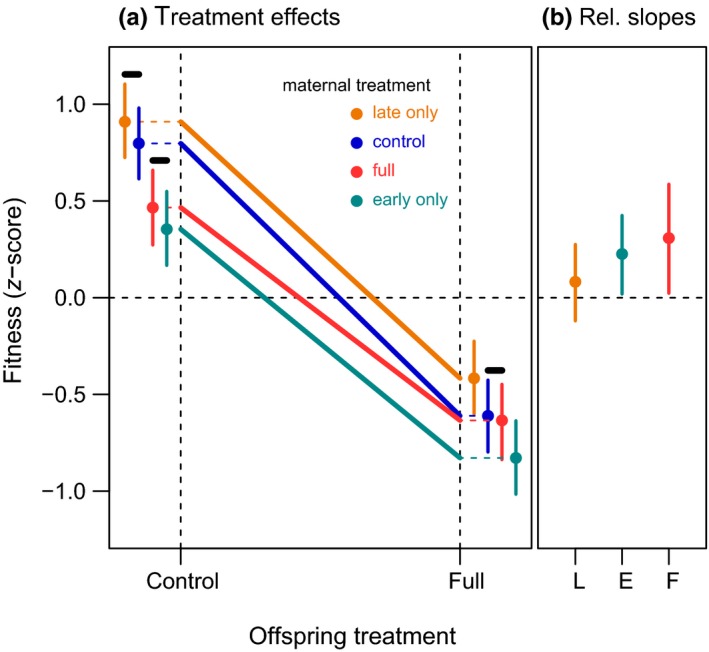
(a) Estimated effects of the maternal treatments on offspring fitness for offspring in control and full exposed treatment. Estimates are constructed from the posterior effect sizes presented in the results section and Figure 2. Marked with a black bar are the treatments which did not differ from each other. In the control offspring treatment, offspring from the late and control maternal treatment performed similarly as did offspring from the full and early maternal treatment. In the fully exposed offspring treatment, offspring from the control and full maternal treatment performed similarly and better than the early, and worse than the late maternal treatment. (b) The slopes (differences in means for the control and full offspring treatment) of Figure 3a relative to the slope of the maternal control treatment. For all treatments offspring in the full exposed treatment do relative better than the control treatment and for offspring of the maternal early (E) and full (F) treatment the 95% credible interval of the difference in slope does not include zero. The improvement for offspring from the maternal late treatment (L) does not differ from zero

Maternal fitness was negatively affected by early maternal exposure (−1.35 *SD*, CI: −1.54, −1.16, effect size 1 in Figure 2b) and less so by late maternal exposure, for which the 95% credible interval of the posterior distribution included zero (−0.10 *SD*, CI: −0.29, 0.083, effect size 2 in Figure 2b). Offspring fitness was negatively affected by offspring exposure (−1.41 *SD*, CI: −1.58, −1.24, effect size 3 in Figure 2b), to a similar extent as the cumulative effect of early and late maternal exposure on maternal fitness (Figure 2b). Early maternal exposure negatively affected offspring fitness (−0.44 *SD*, CI: −0.58, −0.29, effect size 4 in Figure 2b), whereas late maternal exposure had a weak positive effect, for which the 95% credible interval of the posterior distribution included zero (0.11 *SD*, CI: −0.028, 0.26, effect size 5 in Figure 2b). The interaction between early maternal exposure and offspring exposure on offspring fitness was positive (0.23 *SD*, CI: 0.019, 0.43, effect size 6 in Figure 2b). The interaction between late maternal exposure and offspring exposure was weakly positive, but the 95% credible interval of the posterior distribution included zero (0.082 *SD*, CI: −0.12, 0.28, effect size 7 in Figure 2b). The direct and indirect effects explained 58.0% (CI: 52.4, 66.1) of the total amount of variance. While accounting for the direct and indirect effects of the experiments, 5.8% (CI: 1.5, 16.7) of the remaining variance could be attributed to differences between the clone lines. Effects of the maternal treatments on offspring fitness are plotted in Figure 3a; while offspring fitness was reduced under offspring exposure, this fitness decrease was, relative to the maternal control treatment, positive and strongest for the maternal full exposure treatment (0.31 *SD*, CI: 0.023, 0.59, effect size F in Figure 3b), positive and slightly smaller for the maternal early treatment (0.23 *SD*, CI: 0.019, 0.43, effect size E in Figure 3b) and positive but not differing from zero for the maternal late treatment (0.082 *SD*, CI: −0.12, 0.28, effect size L in Figure 3b).

### 3.2 Egg size

**Figure 4:**
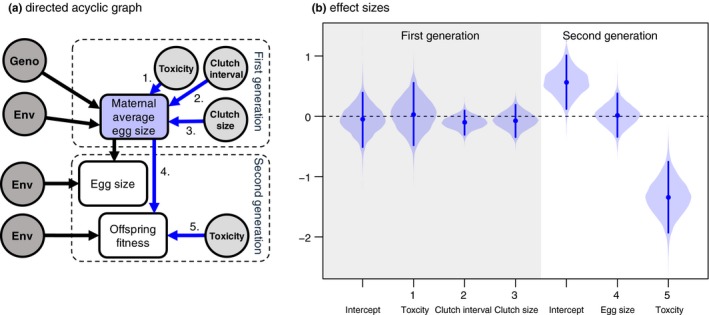
Directed acyclic graph of (a) the model predicting average maternal egg size and its effect on offspring fitness. Colored arrows in (a) and correspond to posterior estimates presented in (b). Dots are the means, whiskers are the 95% credible intervals and violins are the distributions of the posterior estimates. On the gray background are the effects on the maternal phenotype (first generation) and on the white background are the effects in the offspring phenotype (second generation)

Eggs from mothers in a toxic environment were only 2% larger on average than eggs from mothers in the non‐toxic environment and the 95% credible interval of the posterior distribution included zero (0.11 *SD*, CI: −0.26, 0.52, effect size 1 in Figure 4b). Clutch interval (−0.10 *SD*, CI: −0.31, 0.097, effect size 2 in Figure 4b) and clutch size (−0.070 *SD*, CI: −0.35, 0.19, effect size 3 in Figure 4b) did not affect egg size. As in the above model, offspring fitness was strongly negatively impacted by toxic cyanobacteria (−1.34 *SD*, CI: −1.93, −0.75, effect size 4 in Figure 4b), but fitness was not affected by the size of the eggs (0.013 *SD*, CI: −0.34, 0.38, effect size 5 in Figure 4b).

### 3.3 Meta‐analysis

**Figure 5:**
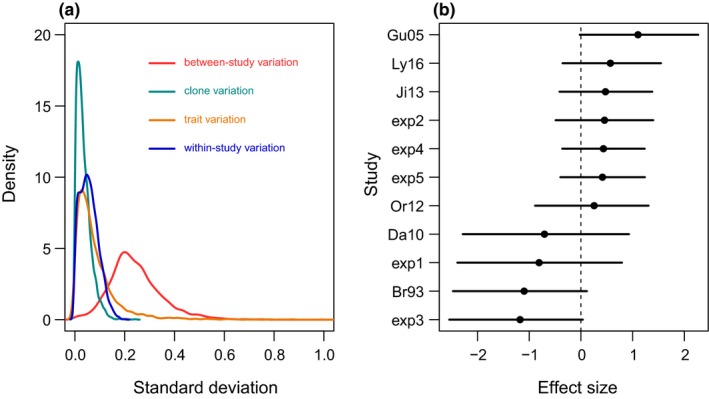
(a) Posterior distributions of the standard deviations of the different sources of variation; namely, between‐study (in red), clone (in green), type of trait (in orange), and within‐study variation (in blue). (b) Per study estimated effect sizes of the maternal effects. Positive values refer to increased fitness for offspring exposed to microcystin if their mothers also were exposed to microcystin. Dots are means and error bars the 95% credible intervals. The experiments in this study are number from exp1 to epx5. Other studies in this meta‐analysis are as follows: Gustafsson et al. (2005; Gu05), Lyu et al. (2016; Ly16), Jiang et al. (2013; Ji13), Ortiz‐Rodriguez et al. (2012; Or12), Dao, Do‐Hong, and Wiegand (2010; Da10), Brett (1993; Br93)

We found six studies reporting a total of 46 effects on nine clones of three species of *Daphnia (D. magna*,* D. longispina*,* D. carinata*). We added data from our five experiments adding 142 effects on 10 clones (traits for experiment 1 were combined for all clone lines, due to small sample sizes). The 95% credible interval of the posterior distribution of the intercept included zero (*µ* = −0.116, CI: −0.335, 0.0875), suggesting that, overall, individuals that are exposed to microcystin do not perform better if their mother was also exposed. Most variation was explained by study (58.5% CI: 24.7, 85.3, Figure 5a), but none of the studies had a 95% credible of its posterior distribution excluding zero (Figure 5b). Trait class explained 18.0% (CI: 0.9, 51.6) and clone 9.1% (CI: 0.3, 28.9) of the variation. We did not find an effect of the concentration of microcystin the *Daphnia* were exposed to on offspring tolerance (β = 0.030, CI: −0.0588, 0.144).

## 4 DISCUSSION

Organisms that encounter stressful conditions will often respond in ways that ameliorate some of the negative effects. Repeated exposure over evolutionary time should make organisms increasingly able to mount precise responses, often relying on environmental cues that can be detected before encountering the stressor. Classic cases of such adaptive plasticity (or predictive adaptive responses; Gluckman & Hanson, 2004) include seasonal morphs, where a photoperiod or temperature cue elicit alternative developmental trajectories that result in locally adapted phenotypes (e.g., in various insect species; Simpson, Sword, & Lo, 2011). Evolution of maternal effects is subject to similar considerations; counteracting negative effects by anticipating stressful conditions experienced by the offspring (Uller, 2008). Systems that have evolved to transfer information about local conditions from parents to offspring should therefore have properties that increase the amount of information transferred to offspring, while reducing any negative side effects of maternal exposure.

Here, we showed that offspring from mothers exposed to toxic cyanobacteria were able to partly compensate for the negative effects of being exposed themselves. However, despite the regular seasonal exposure to toxic cyanobacteria in our study population (Gustafsson, 2007; Schwarzenberger et al., 2013), the positive effect of maternal exposure on offspring tolerance (or resistance) was small relative to the negative fitness effects. Furthermore, rather than being elicited in response to toxic cyanobacteria during reproduction, these positive effects on offspring fitness under exposure were only born out when mothers were exposed early in life. The overall weak effect size we observed was further supported by our meta‐analysis of 46 effects across six separate studies, which also showed lack of general support for individuals receiving benefits when mothers were exposed to toxic cyanobacteria.

Maternal exposure early in life had a positive effect on offspring fitness in the toxic environment, while the effect of maternal exposure during reproduction did not differ from zero. Thus, although mothers exposed to microcystin during growth appear to transfer some tolerance or resistance to offspring, the mechanism does not appear particularly well designed for transmitting information about the presence of microcystin. We therefore suggest that both positive and negative effects of maternal exposure largely reflect passive consequences of females reproducing when they themselves have acquired tolerance, for example, through non‐regulated transference of metabolites to eggs or developing offspring. It has been suggested that such passive maternal effects can represent an early evolutionary stage in the transition from stress‐induced parental effects to local adaptation (Badyaev, 2009; Badyaev & Uller, 2009; Uller, 2012). However, *Daphnia* may be stuck in this state if neither anticipatory maternal effects nor genetic fixation of tolerance is possible because of the fitness costs of developing a tolerant phenotype during periods of the year when cyanobacteria are absent.

More robust inference about the extent to which the maternal effects demonstrated here have in fact been fine‐tuned to transmit information would require a similar experiment on *Daphnia* from populations without an evolutionary history of cyanobacteria exposure (e.g., Badyaev, 2009). Furthermore, we should not rule out the possibility that the magnitude of both negative and positive effects on offspring tolerance in experiments like ours fail to represent the situation in nature. More generally, our study emphasizes that it can be difficult to isolate the adaptive significance of maternal effects without several additional experimental treatments in addition to the standard fully factorial design (Engqvist & Reinhold, 2016). The fact that our study suggests that the overall maternal effect consists of both positive and negative effects might explain the weak and inconclusive findings of previous studies, since the strength of the positive and negative effects may vary depending on experimental design, populations or clone identity.

What kind of mechanism could be responsible for maternal effects in the presence of microcystin‐producing cyanobacteria? *Daphnia* that are exposed to microcystin downregulate transporter genes which are involved in the transport of secondary metabolites (such as microcystin). Downregulation of those genes suggests *Daphnia* improves tolerance to microcystin by reducing the uptake of microcystin, at the cost of reducing the uptake of other metabolites (Schwarzenberger et al., 2014). Many species of cyanobacteria also produce protease inhibitors, which inhibit digestive proteases such as trypsins and chymotrypsins in the *Daphnia* gut. *Daphnia* respond by increasing the production of those enzymes to maintain their capacity for protein digestion (Schwarzenberger, Zitt, Kroth, Mueller, & Elert, 2010), which seems to be realized by the upregulation of genes involved in protein uptake and synthesis (Asselman et al., 2012; Schwarzenberger & Fink, 2018).

The negative consequences of microcystin on offspring fitness are therefore likely to be a consequence of impaired maternal nutrient uptake and metabolism that result in smaller adults with poor energy state, and eggs of a lower nutritional value. The positive maternal effects on offspring fitness could be due to a number of different mechanisms, several of which would be consistent with the scenario of passive transfer of tolerance discussed above. In particular, a recent study demonstrated that the gut microbiota is an important component of *Daphnia* tolerance to cyanobacteria (Macke, Callens, Meester, & Decaestecker, 2017). Since prolonged ingestion should result in a more thorough replacement of microbiota (Macke et al., 2017), this mechanism may explain why exposure during reproduction alone does not induce a positive effect in offspring. Furthermore, microbiota may be passively transmitted to offspring either during early development, while in the brood pouch, or even following birth through the water. The latter implies that, in natural populations, maternal exposure will be less important than the microbial community in the water. Other mechanisms are also possible, however, including changes in egg composition or various forms of epigenetic inheritance that results in upregulation of genes known to be involved in the detoxification of ingested cyanobacteria (Schwarzenberger & Von Elert, 2013).

In summary, maternal exposure to microcystin‐producing cyanobacteria can result in increased offspring tolerance to microcystin. However, the timing of maternal effects, their magnitude relative to the negative fitness consequences for offspring, and weak overall support across studies shed doubt on their adaptive function. We therefore suggest that this maternal effect represents an example of how phenotypic accommodation to stressful conditions can span generations, rather than a mechanism that has been selected for its ability to transmit information from parents to offspring.

## AUTHORS’ CONTRIBUTIONS

RR, AH, DWAN, and TU designed the study. RR and AH collected the data. RR carried out the statistical analyses. RR, AH, DWAN, and TU interpreted the data. RR and TU wrote the manuscript with input from AH and DWAN. All authors gave final approval for publication.

## DATA ACCESSIBILITY

Data are accessible from Dryad (https://doi.org/10.5061/dryad.9j5q334). Stan code of the models is available in Appendices S1–S5.
