# Supplementary material for: Timing of maternal exposure to toxic cyanobacteria and offspring fitness in Daphnia magna: Implications for the evolution of anticipatory maternal effects
Source: Ecol Evol. 2018 Nov 20;8(24):12727–36. doi: 10.1002/ece3.4700 (PMC6309005; doi:10.1002/ece3.4700)
Supplement: Supplementary file 2 [file ECE3-8-12727-s002.docx]

**Table S2:** Cross-validation of maternal effect models explaining total number of offspring. The covariates included in the models are marked with X. The full model is marked in grey. Values between brackets are the standard errors. $\hat{p}$ is the estimated effective number of parameters, which is the same for WAIC and LOOIC. WAIC is the Watanabe-Akaike Information Criterion and is more appropriate for selecting models to predict new observations under the given parameters (e.g. for the presented genotypes). LOOIC is the approximate leave-one-out information criterion and is appropriate to predict new observations under new sets of parameters (e.g., different genotypes). WAIC and LOOIC values are up to the first decimal similar. Cross-validation is based on the difference between the expected log pointwise predictive density for a new dataset (elpd) with the elpd of the best model for both WAIC and LOOIC ($\Delta\hat{elpd}_{waic}$ and $\Delta\hat{elpd}_{loo}$ respectively). Models are considered to perform similarly in predicting new data sets when the log pointwise predictive density is smaller than 1.96 times its standard error. Only models which were performing similarly to the best model are presented in the table as well as the full model, which performs slightly less than the best model.

| Covariates | | | | | | | Cross-validation | | | | |
| --- | --- | --- | --- | --- | --- | --- | --- | --- | --- | --- | --- |
| Mother | | Offspring | | | | | $\hat{p}$ | WAIC | $\Delta\hat{elpd}_{waic}$ | LOOIC | $\Delta\hat{elpd}_{loo}$ |
| Early | Late | Full | Early | Late | Full x early | Full x late |  |  |  |  |  |
| X | X | X | X | X | X |  | 14.0 (0.8) | 2474.9 (50.9) | N.A. | 2474.9 (50.9) | N.A. |
| X | X | X | X |  | X | X | 13.6 (0.8) | 2475.5 (51.1) | 0.3(1.5) | 2475.5 (51.1) | 0.3(1.5) |
| X | X | X | X | X | X | X | 14.6 (0.8) | 2476.1 (51.0) | 0.6(0.3) | 2476.1 (51.0) | 0.6(0.3) |
| X | X | X | X |  | X |  | 13.2 (0.8) | 2476.6 (51.0) | 0.9(1.9) | 2476.6 (51.0) | 0.9(1.9) |
| X |  | X | X | X | X |  | 12.9 (0.7) | 2477.7 (51.2) | 1.4(2.3) | 2477.7 (51.2) | 1.4(2.3) |
| X |  | X | X |  | X | X | 12.8 (0.7) | 2479.0 (51.2) | 2.1(2.8) | 2479.0 (51.2) | 2.1(2.8) |
| X |  | X | X |  | X |  | 12.1 (0.7) | 2479.7 (51.2) | 2.4(3.0) | 2479.7 (51.2) | 2.4(3.0) |
| X |  | X | X | X | X | X | 13.9 (0.8) | 2479.7 (51.2) | 2.4(2.3) | 2479.7 (51.2) | 2.4(2.3) |
| X | X | X | X | X |  |  | 12.9 (0.7) | 2487.8 (50.4) | 6.5(3.9) | 2487.8 (50.4) | 6.5(3.9) |
| X | X | X | X |  |  | X | 12.6 (0.7) | 2488.5 (50.4) | 6.8(4.1) | 2488.5 (50.4) | 6.8(4.1) |
| X | X | X | X |  |  |  | 11.8 (0.7) | 2489.3 (50.3) | 7.2(4.3) | 2489.3 (50.3) | 7.2(4.3) |
| X | X | X | X | X |  | X | 13.8 (0.8) | 2489.8 (50.3) | 7.5(3.9) | 2489.8 (50.3) | 7.5(3.9) |
| X |  | X | X | X |  |  | 12.0 (0.7) | 2491.0 (50.6) | 8.1(4.5) | 2491.0 (50.6) | 8.1(4.5) |
| X |  | X | X |  |  | X | 11.7 (0.7) | 2491.6 (50.7) | 8.4(4.7) | 2491.6 (50.7) | 8.4(4.7) |
| X |  | X | X |  |  |  | 11.0 (0.6) | 2492.6 (50.6) | 8.9(4.9) | 2492.6 (50.6) | 8.9(4.9) |
| X |  | X | X | X |  | X | 13.1 (0.7) | 2493.1 (50.6) | 9.1(4.5) | 2493.1 (50.6) | 9.1(4.5) |
