# Supplementary material for: Timing of maternal exposure to toxic cyanobacteria and offspring fitness in Daphnia magna: Implications for the evolution of anticipatory maternal effects
Source: Ecol Evol. 2018 Nov 20;8(24):12727–36. doi: 10.1002/ece3.4700 (PMC6309005; doi:10.1002/ece3.4700)
Supplement: Supplementary file 3 [file ECE3-8-12727-s003.pdf]

Supporting Information for:

Timing of maternal exposure to toxic cyanobacteria and offspring fitness in *Daphnia magna*: implications for the evolution of anticipatory maternal effects

Reinder Radersma, Alexander Hegg, Daniel W.A. Noble, Tobias Uller

Submitted to *Ecology and Evolution*

Index **Page**

Index	Page
Appendix S1: Methods – Model descriptions	2
Appendix S2: Stan code for maternal effects model	6
Appendix S3: Stan code for egg size measurement model	8
Appendix S4: Stan code for meta-analysis model without covariates	10
Appendix S5: Stan code for meta-analysis model with toxicity as covariate	11
Figure S1	12

Appendix S1: Methods – Model descriptions

The models to estimate the maternal effects, egg sizes and related fitness and the model for meta-analysis are described below in full matrix notation. The design matrices for the fixed effects, notated as $\boldsymbol{X}$ as well as the poster estimated effect sizes $\boldsymbol{\beta}$, varied for the maternal effect and meta-analysis model depending on the covariates in the model. In the design matrix for the fixed effects $\boldsymbol{X}$ each column contained the values for one covariate, with the first column containing only 1's for the intercept. In the design matrix for the random effects $\boldsymbol{Z}$ each column represents one class, for which 1 means the observation was within this class and 0 means it was not. All rows of $\boldsymbol{Z}$ sum to 1.

Matrices are written as bold upper case Latin letters, vectors are bold lower case Latin or Greek letters, and scalars are non-bold lower case Latin or Greek letters. Latin letters indicate observed variables, while Greek letters indicate estimated parameters. Superscript is used to discriminate between matrices and vectors containing similar information. To reduce the use of superscripts, parameter symbols are reused in each paragraph, describing a different model.

Maternal effects model

The maternal effects model consists of two general linear mixed models, which have their random effects jointly estimated. The maternal fitness $\boldsymbol{y^M}$ was sampled from the following normal distribution;

$$\boldsymbol{y^M} \sim N\left(\boldsymbol{X^M}\boldsymbol{\beta^M} + \boldsymbol{Z^M}\boldsymbol{\delta}, \sigma\right), \qquad (1)$$

in which $\boldsymbol{X^M}$ is the design matrix for the fixed effects, $\boldsymbol{\beta^M}$ is the vector of posterior fixed effect sizes, $\boldsymbol{Z^M}$ Is the design matrix for the genetic effects, $\boldsymbol{\delta}$ is the vector of genetic effects for each genotype and with a standard deviation of σ. The offspring fitness $\boldsymbol{y^O}$ was sampled from the following normal distribution;

$$\boldsymbol{y^O} \sim N\left(\boldsymbol{X^O}\boldsymbol{\beta^O} + \boldsymbol{Z^O}\boldsymbol{\delta}, \sigma\right), \tag{2}$$

in which $\boldsymbol{X^O}$ is the design matrix for the fixed effects, $\boldsymbol{\beta^O}$ is the vector of posterior fixed effect sizes and $\boldsymbol{Z^O}$ is the design matrix for the genetic effects. $\boldsymbol{\delta}$ is sampled from a normal distribution,

$$\boldsymbol{\delta} \sim N(0, \tau), \tag{3}$$

with a mean of zero and a standard deviation τ. $\boldsymbol{\beta^M}$ and $\boldsymbol{\beta^O}$ were drawn from a uniform distribution from -10 to 10 and σ and τ were drawn from a half-Cauchy distribution with a mean of zero and a scale parameter of 0.1. For all models, we ran 4 chains of 10,000 iterations, of which we discarded the first 5,000 iterations as burn-in and thinned the iteration chains by only storing every tenth iteration.

Egg size models

The egg size measurements $\boldsymbol{y^E}$ were sampled from the following normal distribution;

$$\boldsymbol{y^E} \sim N(\boldsymbol{Z^E}\boldsymbol{\mu}, \sigma) \tag{4}$$

in which $\boldsymbol{Z^E}$ is the design matrix of the maternal identity, $\boldsymbol{\mu}$ is the average egg size produced by the mothers and with a standard deviation of σ. $\boldsymbol{\mu}$ is sampled from a normal distribution,

$$\boldsymbol{\mu} \sim N(\boldsymbol{X^E}\boldsymbol{\beta^E} + \boldsymbol{Z^G}\boldsymbol{\delta}, \tau) \tag{5}$$

in which $\boldsymbol{X^E}$ is the design matrix, $\boldsymbol{\beta^E}$ is the vector of posterior effect sizes, $\boldsymbol{Z^G}$ is the design matrix for the genetic effects, $\boldsymbol{\delta}$ is the vector of genetic effects for each genotype and with a standard deviation of τ. $\boldsymbol{\delta}$ is sampled from a normal distribution,

$$\boldsymbol{\delta} \sim N(0, \upsilon) \tag{6}$$

with a mean of zero and a standard deviation υ. The fitness measurements $\boldsymbol{y^F}$ was sampled from the following normal distribution;

$$\boldsymbol{y^F} \sim N(\boldsymbol{X^F}\boldsymbol{\beta^F} + \boldsymbol{Z^F}\boldsymbol{\mu}, \varphi) \tag{7}$$

in which $\boldsymbol{X^F}$ is the design matrix, $\boldsymbol{\beta^F}$ is the vector of posterior effect sizes, $\boldsymbol{Z^F}$ Is the design matrix for the maternal egg size effects and with a standard deviation of φ. $\boldsymbol{\beta^E}$ and $\boldsymbol{\beta^F}$ were

drawn from a uniform distribution from -10 to 10 and σ, τ, υ and φ were drawn from a half-Cauchy distribution with a mean of zero and a scale parameter of 0.1. For all models, we ran 4 chains of 150,000 iterations, of which we discarded the first 50,000 iterations as burn-in and thinned the iteration chains by only storing every hundredth iteration.

Models for meta-analysis

For the meta-analysis, we used a multi-level meta-analytic model (Nakagawa & Santos, 2012). The effect size estimates $\boldsymbol{y}$ were drawn from the following distribution;

$$\boldsymbol{y} \sim N\left(\boldsymbol{X\beta} + \boldsymbol{Z^H\gamma} + \boldsymbol{Z^C\delta} + \boldsymbol{Z^T\zeta} + \boldsymbol{\eta}, \boldsymbol{s}_{\bar{y}}\right) \tag{8}$$

in which $\boldsymbol{X}$ is the fixed effects design matrix, $\boldsymbol{\beta}$ is the vector of posterior fixed effect sizes, $\boldsymbol{\gamma}$ are the between-study effects with their corresponding design matrix $\boldsymbol{Z^H}$, $\boldsymbol{\delta}$ are the between-clone effects with their corresponding design matrix $\boldsymbol{Z^C}$, $\boldsymbol{\zeta}$ are the trait-class effects with their corresponding design matrix $\boldsymbol{Z^T}$, $\boldsymbol{\eta}$ are the within-study effects and $\boldsymbol{s}_{\bar{y}}$ are the standard errors for the effect size estimates (i.e., sampling variance). $\boldsymbol{\gamma}$ – the between-study effects – are drawn from a normal distribution with standard deviation τ, the between study heterogeneity;

$$\boldsymbol{\gamma} \sim N(0, \tau) \tag{9}$$

$\boldsymbol{\delta}$ – the between-clone effects – are drawn from a normal distribution with standard deviation υ, the clone variability;

$$\boldsymbol{\delta} \sim N(0, \upsilon) \tag{10}$$

$\boldsymbol{\zeta}$ – the trait-class effects – are drawn from a normal distribution with standard deviation φ, the trait-class variability;

$$\boldsymbol{\zeta} \sim N(0, \varphi) \tag{11}$$

$\boldsymbol{\eta}$ – the within-study effects – are drawn from a normal distribution with standard deviation χ, the within-study variability;

$$\boldsymbol{\eta} \sim N(0, \chi) \tag{12}$$

We first ran the model with only an intercept as fixed effect. Next, we ran the model with an intercept and the concentration of microcystin used in the studies as fixed effects. $\boldsymbol{\beta}$ were drawn from a uniform distribution from -10 to 10 and σ, τ, υ, φ and χ were drawn from a half-Cauchy distribution with a mean of zero and a scale parameter of 0.1. For all models, we ran 4 chains of 12,000 iterations, of which we discarded the first 2,000 iterations as burn-in and thinned the iteration chains by only storing every tenth iteration.

References

Nakagawa, S. & Santos, E.S.A. 2012. Methodological issues and advances in biological meta-analysis. *Evol. Ecol.* **26**: 1253–1274.

Appendix S2: Stan code for maternal effects model

```
data {
  int<lower=0>    nci;         // number covariates for offspring
  int<lower=0>    ncm;         // number covariates for mothers
  int<lower=0>    ni3;         // number of individuals exp 3
  matrix[ni3,nci] Ci3;         // matrix with covariates for individuals in exp 3
  int<lower=1>    gi3[ni3];    // genotypes of individuals exp 3

  int<lower=0>    nm3;         // number of mothers exp 3
  matrix[nm3,ncm] Cm3;         // matrix with covariates for mothers in exp 3
  int<lower=1>    gm3[nm3];    // genotypes of mothers exp 3

  vector[ni3+nm3] ph3;         // phenotype for mothers and individuals exp 3

  int<lower=0>    ni5;         // number of individuals exp 5
  matrix[ni5,nci] Ci5;         // matrix with covariates for individuals in exp 5
  int<lower=1>    gi5[ni5];    // genotypes of individuals exp 5

  int<lower=0>    nm5;         // number of mothers exp 5
  matrix[nm5,ncm] Cm5;         // matrix with covariates for mothers in exp 5
  int<lower=1>    gm5[nm5];    // genotypes of mothers exp 5

  vector[ni5+nm5] ph5;         // phenotype for mothers and individuals exp 5
  }

parameters {
  vector<lower=-5,upper=5>[nci] beta_i;  // effect sizes for individuals
  vector<lower=-5,upper=5>[ncm] beta_m;  // effect sizes for mothers
  vector<lower=-5,upper=5>[7]   gamma;   // breeding values
  real<lower=0>                 sigma_G; // additive genetic variance
  real<lower=0>                 sigma_E; // environmental variance
  }

transformed parameters {
  vector[ni3+nm3] mu3;
  vector[ni5+nm5] mu5;

  for(i in 1:nm3)
    mu3[i]     = dot_product(beta_m,Cm3[i,]) + gamma[gm3[i]];

  for(i in 1:ni3)
    mu3[i+nm3] = dot_product(beta_i,Ci3[i,]) + gamma[gi3[i]];

  for(i in 1:nm5)
    mu5[i]     = dot_product(beta_m,Cm5[i,]) + gamma[gm5[i]];

  for(i in 1:ni5)
    mu5[i+nm5] = dot_product(beta_i,Ci5[i,]) + gamma[gi5[i]];
  }

model {
  // individual environment effects
  target += normal_lpdf(ph3 | mu3, sigma_E);
  target += normal_lpdf(ph5 | mu5, sigma_E);

  // additive genetic values
  target += normal_lpdf(gamma | 0, sigma_G);
```

```
  // standard deviations
  target += cauchy_lpdf(sigma_G | 0, 0.1);
  target += cauchy_lpdf(sigma_E | 0, 0.1);
  }

generated quantities {
  real log_lik[nm3+ni3+nm5+ni5];
  for(i in 1:(nm3+ni3))
    log_lik[i] = normal_lpdf(ph3[i] | mu3[i], sigma_E);
  for(i in 1:(nm5+ni5))
    log_lik[nm3+ni3+i] = normal_lpdf(ph5[i] | mu5[i], sigma_E);
  }
```

Appendix S3: Stan code for egg size measurement model

```
data {
  int<lower=0>           ns;        // number of egg size measurements
  int<lower=0>           nf;        // number of fitness estimates
  int<lower=0>           ng;        // number of genotypes
  int<lower=0>           nm;        // number of mothers
  real                   ph_s[ns];  // egg size measurements offspring
  real                   ph_f[nf];  // fitness measurements offspring
  int<lower=0, upper=1>  txf[nf];   // toxicity offspring environment
  real                   dev[nm];   // development time
  real                   bsz[nm];   // average of brood size 2 + 3
  int<lower=0, upper=1>  tox[nm];   // number of successes
  int<lower=1,upper=ng>  gid[nm];   // genetic identity of mother
  int<lower=1,upper=nm>  mid_s[ns]; // maternal identity of egg measurement
  int<lower=1,upper=nm>  mid_f[nf]; // maternal identity of fitness estimate
  }

parameters {
  vector[nm] ph_m;
  vector[ng] eta;

  real<lower=-10, upper=10> beta0_f;
  real<lower=-10, upper=10> beta1_f;
  real<lower=-10, upper=10> beta2_f;
  real<lower=-10, upper=10> beta0_m;
  real<lower=-10, upper=10> beta1_m;
  real<lower=-10, upper=10> beta2_m;
  real<lower=-10, upper=10> beta3_m;

  real<lower=0> sigma_s;
  real<lower=0> sigma_f;
  real<lower=0> sigma_m;
  real<lower=0> sigma_g;
  }

transformed parameters{
  vector[ns] mu_s;
  vector[nf] mu_f;
  vector[nm] mu_m;

  for(i in 1:ns)
    mu_s[i] = ph_m[mid_s[i]];
  for(i in 1:nf)
    mu_f[i] = beta0_f + beta1_f * ph_m[mid_f[i]] + beta2_f * txf[i];
  for(i in 1:nm)
    mu_m[i] = beta0_m + beta1_m * tox[i] + beta2_m * dev[i] + beta3_m * bsz[i] +
       eta[gid[i]];
  }

model {
  vector[ng] zeros; // vector with zeros
  zeros = rep_vector(0.0,ng);

  // egg size
  target += normal_lpdf(ph_s | mu_s, sigma_s);

  // fitness estimates
  target += normal_lpdf(ph_f | mu_f, sigma_f);
```

```
// maternal phenotype
target += normal_lpdf(ph_m | mu_m, sigma_m);

// genotypes
target += normal_lpdf(eta | zeros, sigma_g);

// standard deviations
target += cauchy_lpdf(sigma_s | 0, 0.1);
target += cauchy_lpdf(sigma_f | 0, 0.1);
target += cauchy_lpdf(sigma_m | 0, 0.1);
target += cauchy_lpdf(sigma_g | 0, 0.1);
}
```

Appendix S4: Stan code for meta-analysis model without covariates

```
data {
  int<lower=0> J;         //number of datapoints
  int<lower=0> N_s;       //number of studies
  int<lower=0> N_c;       //number of clones
  int<lower=0> N_t;       //number of traits
  real y[J];              //Hedges' g estimates
  real<lower=0> sigma[J]; // standard error of Hedges' g
  int sid[J];             // study id
  int cid[J];             // clone id
  int tid[J];             // trait id
  }

parameters {
  real mu;
  real<lower=0> tau_h;
  real<lower=0> tau_s;
  real<lower=0> tau_c;
  real<lower=0> tau_t;
  real eta_h[J];
  real eta_s[N_s];
  real eta_c[N_c];
  real eta_t[N_t];
  }

transformed parameters {
  real theta[J];
  for(j in 1:J)
  theta[j] = mu + tau_h * eta_h[j]  + tau_s * eta_s[sid[j]]  + tau_c *
eta_c[cid[j]]  + tau_t * eta_t[tid[j]];
  }

model {
  eta_h ~ normal(0,1);
  eta_s ~ normal(0,1);
  eta_c ~ normal(0,1);
  eta_t ~ normal(0,1);
  y ~ normal(theta,sigma);
  }
```

Appendix S5: Stan code for meta-analysis model with toxicity as covariate

```
data {
  int<lower=0> J;         //number of datapoints
  int<lower=0> N_s;       //number of studies
  int<lower=0> N_c;       //number of clones
  int<lower=0> N_t;       //number of traits
  real y[J];              //Hedges' g estimates
  real<lower=0> sigma[J]; // standard error of Hedges' g
  int sid[J];             // study id
  int cid[J];             // clone id
  int tid[J];             // trait id
  real cov[J];            // covariate
  }

parameters {
  real mu;
  real beta;
  real<lower=0> tau_h;
  real<lower=0> tau_s;
  real<lower=0> tau_c;
  real<lower=0> tau_t;
  real eta_h[J];
  real eta_s[N_s];
  real eta_c[N_c];
  real eta_t[N_t];
  }

transformed parameters {
  real theta[J];
  for(j in 1:J)
    theta[j] = mu + beta * cov[j] + tau_h * eta_h[j]  + tau_s * eta_s[sid[j]]  +
tau_c * eta_c[cid[j]]  + tau_t * eta_t[tid[j]];
  }

model {
  eta_h ~ normal(0,1);
  eta_s ~ normal(0,1);
  eta_c ~ normal(0,1);
  eta_t ~ normal(0,1);
  y ~ normal(theta,sigma);
  }
```

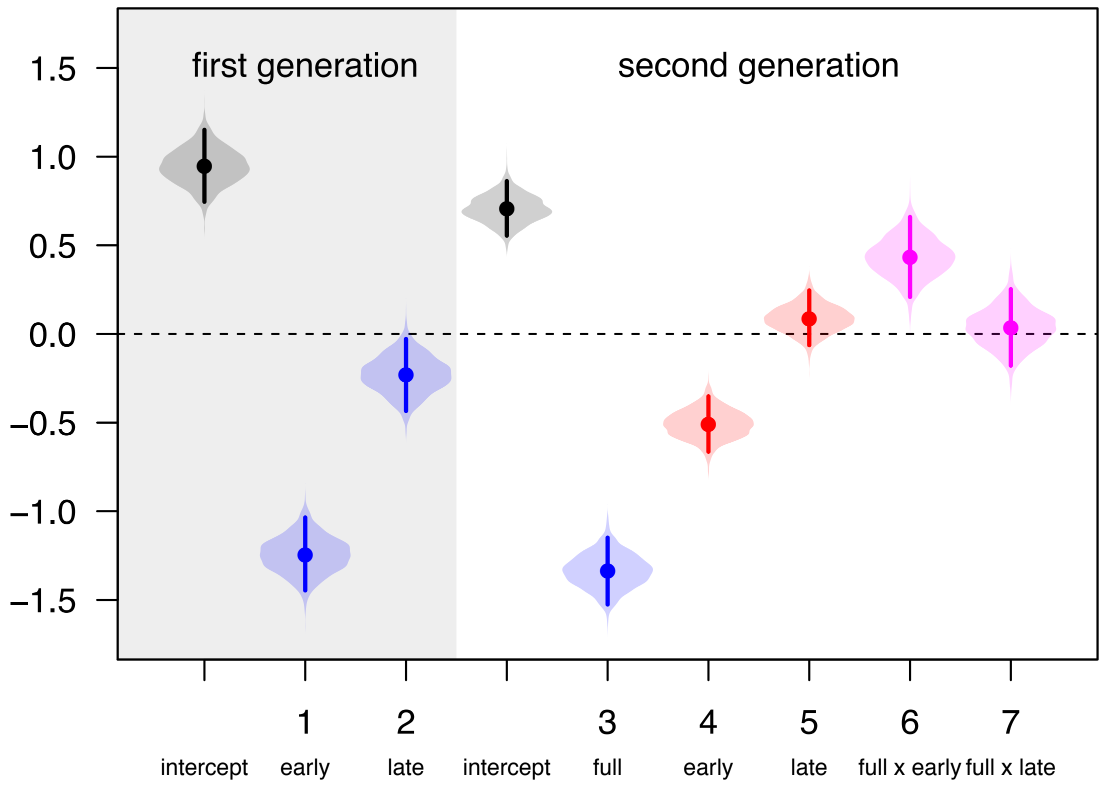

Figure S1. Posterior effect sizes of the full model explaining total number of offspring. Numbers correspond to coloured arrows in Figure 1a. Dots are the means, whiskers are the 95% credible intervals and violins are the distributions of the posterior estimates. In blue are the estimates for direct treatment effects, in red are the estimates for maternal effects and in purple are the interactions of the maternal effects with the offspring environment. On the grey background are the effects on maternal fitness (first generation) and on the white background are the effects on the offspring fitness (second generation). Although the full model was not among the best performing models (see Table S2), it is representative of the best models, in which all covariates are represented by at least 7 out of the 15 models.
